# Did COVID-19 impact Positive Airway Pressure adherence in 2020? A cross-sectional study of 8477 patients with sleep apnea

**DOI:** 10.1186/s12931-022-01969-z

**Published:** 2022-03-04

**Authors:** Fanny Bertelli, Carey M. Suehs, Jean-Pierre Mallet, Isabelle Court-Fortune, Frédéric Gagnadoux, Jean Christian Borel, Olivier Gaubert, Nicolas Molinari, Arnaud Bourdin, Dany Jaffuel

**Affiliations:** 1 Department of Medical Information, Montpellier University Hospital, IDESP, UMR 5149, Montpellier University, 34090 Montpellier, France; 2 Groupe Adène, rue de Chambert, 34080 Montpellier, France; 3 Department of Respiratory Diseases, Univ Montpellier, CHU Montpellier, 371, Avenue Doyen Giraud, 34295 Montpellier Cedex 5, France; 4 PhyMedExp, CNRS, INSERM, Univ Montpellier, CHU Montpellier, Montpellier, France; 5 Department of Respiratory Diseases, CHU Saint Etienne, 25, boulevard Pasteur, 42055 Saint-Étienne Cedex 2, France; 6 Department of Respiratory and Sleep Medicine, Angers University Hospital, INSERM U1063, SOPAM, Angers University, Angers, France; 7 Inserm U1042, HP2 (Hypoxia PhysioPathology), Laboratory Centre Hospitalier Universitaire Grenoble Alpes, Grenoble Alps, University, Grenoble, France

**Keywords:** Sleep Apnea, CPAP, COVID 19, And adherence

## Abstract

**Background:**

Whether the COVID-19 pandemic impacts Positive Airway Pressure (PAP) adherence over the long-term is unknown and only preliminary short-term data have been reported.

**Methods:**

With the aim of describing the impact of the first and second waves of COVID-19 on PAP adherence during 2020 in France, we designed a cross-sectional study of Sleep-Apnea (SA)-patients under PAP telemonitoring. To examine PAP adherence in adult SA patients, we assessed de-identified data from a non-profit healthcare provider database during the period January 1, 2019 to December 31, 2020. Included patients met the following criteria: (i) PAP-treated for at least 4 months before January 1, 2019 and with continuous PAP during both 2019 and 2020; (ii) ≥ 360 daily PAP telemonitored data per year. For PAP adherence, data were collected using the PAP-software.

**Results:**

8477/10482 patients were finally included in the analysis [72.4% male, median age 70 years (IQ_25–75_: 61–77], 25.6% < 62 years old, initial Apnea–Hypopnea Index (AHI) of 41 (31–59)/h. Median PAP adherence was 7.21 (6.12–8.10) h/day in 2020 versus 7.12 (6.05–8.02) h/day in 2019, p < 0.001. The median difference in PAP adherence between the first 2020 lockdown and the corresponding 2019 weeks was 9.75 (CI_95%_ 8.75–10.75) min/day, p < 0.001. The median difference in PAP adherence between the second 2020 lockdown and the corresponding 2019 weeks was 5.00 (CI_95%_ 4.00–6.00) min/day, p < 0.001. If we consider the minimal clinically important difference of 30 min for PAP adherence, 30.4% and 26% of the patients increased their PAP adherence by at least 30 min during the first and second lockdowns respectively; 17.6% and 19.3% of the patients lowered their PAP adherence by at least 30 min in the first and second lockdowns, respectively.

**Conclusion:**

During the first and second lockdowns, the COVID-19 pandemic had a clinically irrelevant effect on PAP adherence for the study population. Future studies are needed to describe COVID-19 pandemic impact on PAP adherence not only for long-term PAP-treated SA patients but also for incident cases.

*Trial registration* The COVADENE study was registered on March 1st, 2021 on ClinicalTrials.gov (Identifier: NCT04775966)

## Background

For sleep apnea (SA) patients, despite advances in alternative therapy, Positive Airway Pressure (PAP) remains the cornerstone of treatment [1], improving daytime sleepiness, sleep quality and quality-of-life proportional to PAP adherence [2, 3].

How the COVID-19 pandemic alters sleep is currently under investigation. Among SA patients, many are at increased-risk for lethal COVID-19 infection due to commonly occurring SA-comorbidities. For PAP-treated patients, additional stress also results from controversial proposals to continue or stop PAP treatment due to the supposed increased risk of COVID-19 infection associated with PAP-flow [4–6].

As recently underlined by a perspective article [7] and a review article [8], whether the COVID-19 pandemic impacts PAP adherence over the long-term is unknown and only preliminary data on the initial 4 weeks of the first lockdown have been reported [9–11]. With the aim of describing the impact of the first and second waves of COVID-19 on PAP adherence during 2020 in France, we designed a cross-sectional study of SA patients under PAP telemonitoring and compared 2020 versus 2019 PAP adherence.

## Methods

### Study design

The COVADENE study was approved by the Montpellier University Hospital Institutional Review Board (reference number IRB-MTP_2021_01_202100723) and registered on ClinicalTrials.gov (NCT04775966). To examine PAP adherence in adult SA patients, we assessed de-identified data from a non-profit healthcare provider database (ADENE group) during the period January 1, 2019 to December 31, 2020. Included patients met the following criteria: (i) treated for SA syndrome (i.e., an initial polygraphy or polysomnography Apnea–Hypopnea Index (iAHI) ≥ 30/h or iAHI ≥ 15/h (and more than 10/h respiratory-effort-related arousal or cardio-metabolic/respiratory comorbidities) associated with three symptoms among sleepiness, tiredness, snoring, headaches, hypertension, reduced vigilance, libido disorders, nocturia, choking or suffocation during sleep); (ii) PAP-treated for at least 4 months before January 1, 2019 and with continuous PAP during both 2019 and 2020; (iii) acceptance of PAP telemonitoring with consent given for data collection and anonymization; (iv) ≥ 360 daily PAP tele monitored data per year for both 2019 and 2000. Using electronic records, information on age, sex, and initial AHI was collected. For PAP adherence, data were collected using the PAP-software. PAP adherence was expressed in hours/day and a PAP adherent patient was defined as a patient with a PAP adherence ≥ 4 h/day per week.

### Statistical analyses

For statistical analyses, continuous data were presented as medians with their associated quartile ranges due to non-Gaussian distributions. Qualitative parameters were presented as numbers and percentages. Continuous variables were compared using Wilcoxon tests (for paired data), and qualitative ones using McNemar tests.

Multivariable logistic regression analyses (MLRA) were used to study associations between explanatory variables [age < 62 years (reflecting the age of professional retirement in France), sex (women) and iAHI ≥ 30/h] and two variables of interest (PAP adherence increase by at least 30 min and PAP adherence decrease by at least 30 min, PAP adherence measured in the first or second lockdown weeks minus the corresponding 2019 weeks). The explanatory variables were systematically fed into multivariable analyses. The two variables of interest were chosen because of the minimal clinically important difference (MCID) of 30 min for PAP adherence [1]. All statistical analyses and figures were performed using R Statistical Software (version 4.0.2 R Foundation for Statistical Computing, Vienna, Austria).

## Results

Of the 10,482 patients in the database, 8477 patients respecting the inclusion criteria were finally analysed. The 8477 patients (72.4% male) had a median age of 70 years (IQ_25–75_: 61–77), 25.6% were < 62 years old, and the median AHI was 41 (31–59)/h.

**Fig. 1:**
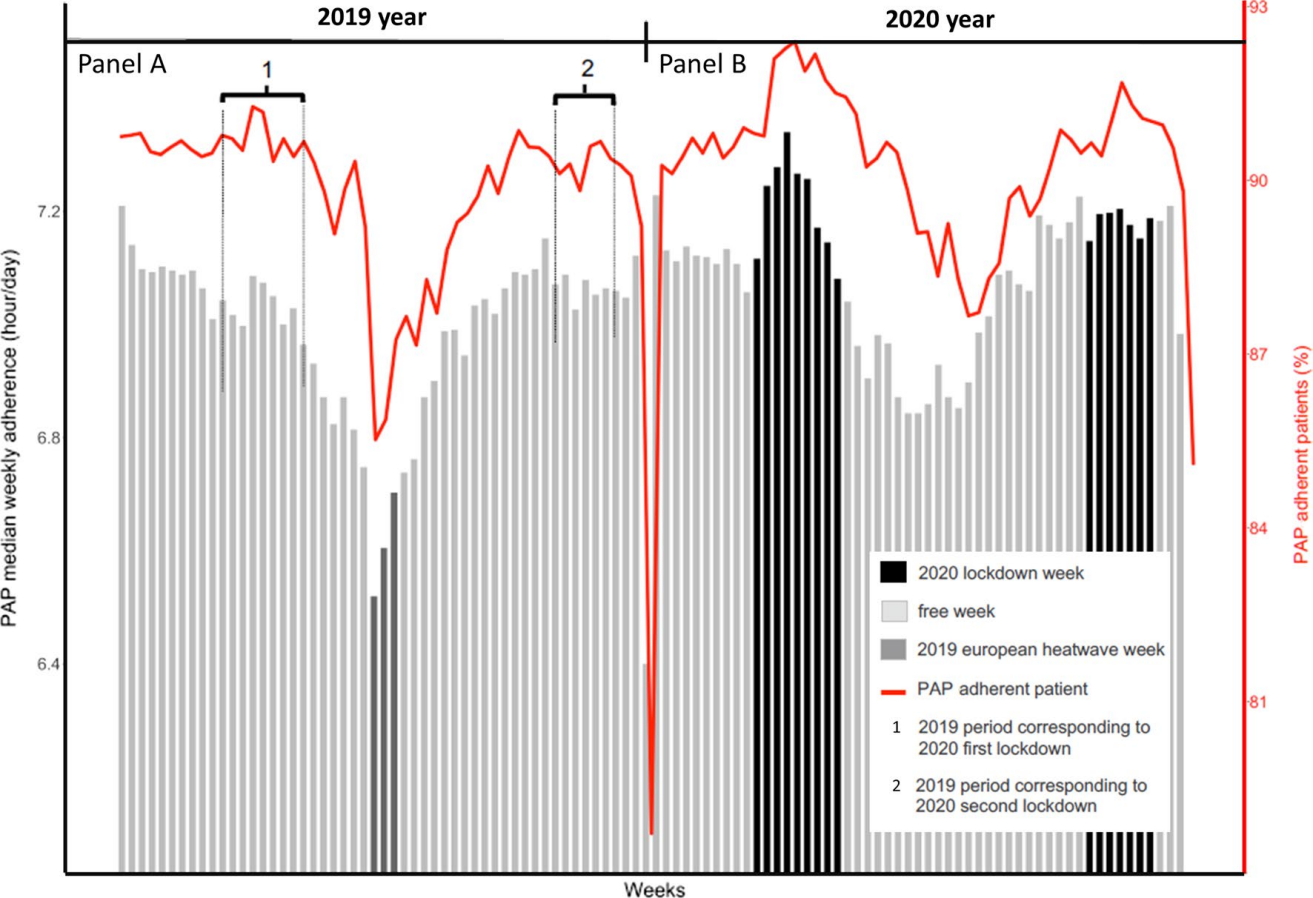
Median PAP adherence (h/day) per week and the proportion of adherent patients per week for the year 2019 (**A**) and 2020 (**B**)

Figure 1 depicts the median PAP adherence (h/day) per week and the median proportion of adherent patients per week for 2019 (panel A) and 2020 (panel B). Median PAP adherence was 7.21 (6.12–8.10) h/day in 2020 versus 7.12 (6.05–8.02) h/day in 2019, p < 0.001. The median difference in PAP adherence between the first 2020 lockdown and the corresponding 2019 weeks was 9.75 (CI_95%_ 8.75–10.75) min/day, p < 0.001. The median difference in PAP adherence between the second 2020 lockdown and the corresponding 2019 weeks was 5.00 (CI_95%_ 4.00–6.00) min/day, p < 0.001. Baseline variability in PAP adherence was measured for the first 4 weeks of each year. A difference (2020 − 2019) was observed with a median deviation of 1.50 min (CI_95%_ 0.50; 2.50), (p = 0.008).

If we consider the MCID of 30 min for PAP adherence [1], 30.4% and 26.0% of the patients increased their PAP adherence by at least 30 min during the first and second lockdowns, respectively, in comparison with their analogous 2019-weeks. Multivariable logistic regression analysis (MLRA) suggests: (i) for the first lockdown, only “age < 62 years” was considered a significant explanatory variable (odds ratio (OR) 1.68 (1.51–1.86), p < 0.001; (ii) for the second lockdown, “age < 62 years” and “women” remained significant [OR 1.30 (1.17–1.45), p < 0.001, OR 1.29 (1.16–1.43), p < 0.001, respectively].

**Fig. 2:**
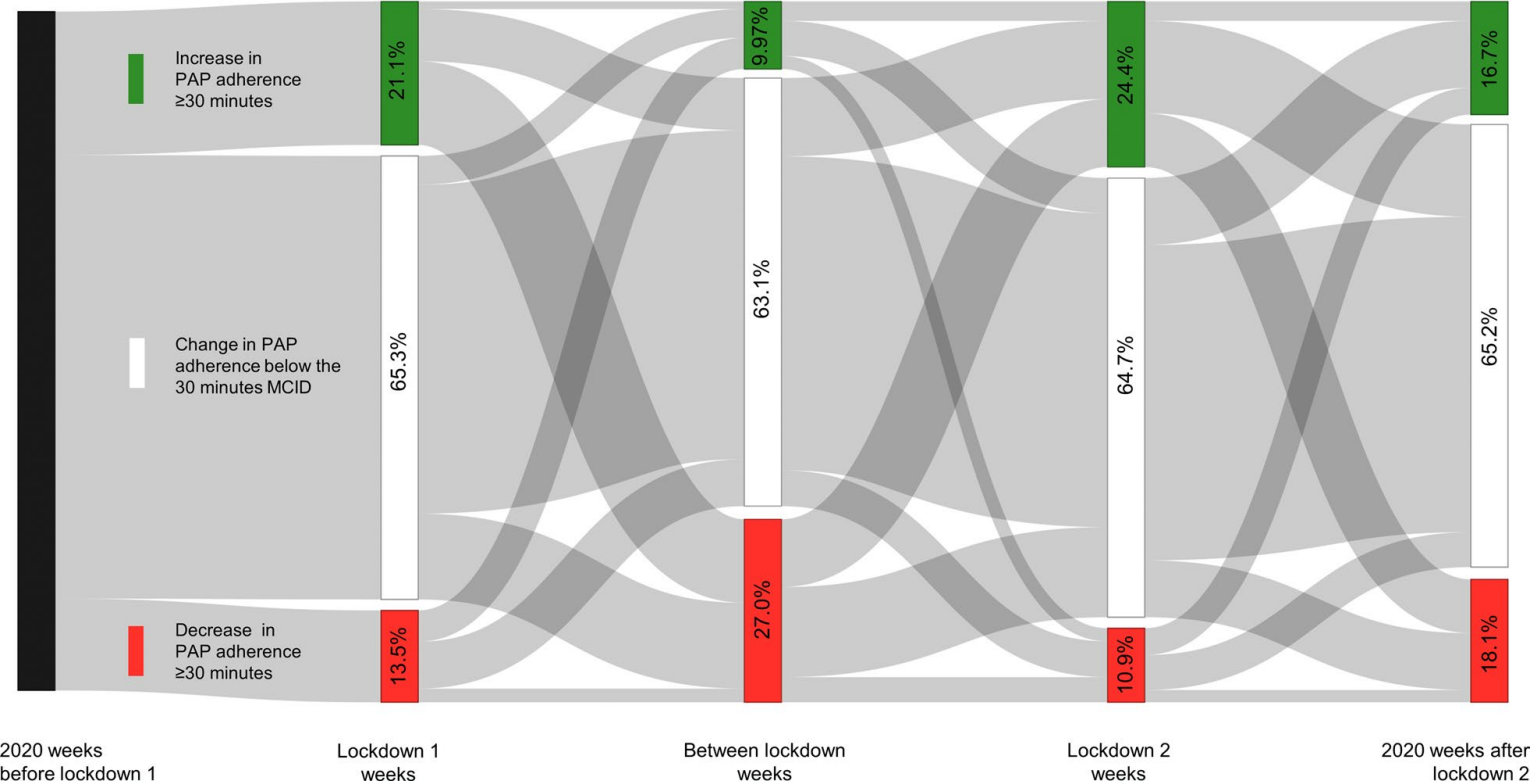
Sankey diagram for the minimal clinically important difference (MCID) of 30 min for PAP adherence in 2020 versus corresponding 2019 periods

On the contrary, 17.6% and 19.3% of the patients lowered their PAP adherence by at least 30 min in the first and second lockdowns, respectively, in comparison with their analogous 2019-weeks. MLRA suggests that only “women” remained a significant explanatory variable for the first lockdown [OR = 1.48 (1.31–1.67), p < 0.001), while women (OR = 1.13 (1.00–1.27), p = 0.040) and age < 62 years (OR = 0.87 (0.77–0.99), p = 0.033] remained for the second lockdown. Figure 2 depicts a Sankey diagram for the MCID trajectories in 2020 and particularly during the lockdown weeks.

The percentage of patients with a median adherence > 4 h/day was 93.43% in 2020 and 93.37% in 2019. The percentages of PAP adherent patients during the first and second lockdowns were not significantly higher than in the corresponding 2019 weeks (with differences of 0.52% and 0.18%, p = 0.0692 and p = 0.5538, respectively). Considering the 12,038 patients treated with PAP for at least 4 months on January 1, 2019, 5.36% stopped PAP therapy in 2019 versus 4.10% in 2020 (p < 0.001). In addition, 188 patients died (1.56%) in 2019 versus 211 (1.88%) in 2020 (p = 0.007).

Other circumstantial events, such as the 2019 heat wave in Europe and 2019/2020 New Year celebrations, impact PAP adherence. The median difference in PAP adherence between the 2020 weeks and the corresponding 2019 heat wave weeks was 11 min/day (− 18 min and 45 min for Q1 and Q3, respectively). The median difference in PAP adherence between the 2020 and 2019 New Year celebration days was 3 min (− 32.5 min and 44 min for Q1 and Q3 respectively).

## Discussion

To our knowledge, this study is the first to describe the impact of the COVID-19 pandemic on the PAP adherence of SA patients for the entire 2020 year. In contrast with three previous studies reporting on only the 4 first weeks of the first lockdown [9–11], we report a two-phase curve for PAP adherence on the full first lockdown duration. As a consequence, we report only a 1.44% median increase (p < 0.001) in PAP adherence (6.25 min) for the full first lockdown in comparison with the 4 weeks preceding the lockdown.

In the COVADENE study, we report a median increase of 5 min (p < 0.001) when comparing PAP adherence between 2020 and 2019. It is important to underline that: (i) for the whole population, this statistically significant difference is largely under the MCID of 30 min [1]; (ii) this difference is not exclusively related to the COVID-19 pandemic but also the consequence of circumstantial events like the 2019 heat wave in Europe. PAP adherence is known to worsen during the summer [12] and we can reasonably speculate that heat waves do not create favorable conditions for PAP adherence. In our opinion, the increase in 2020 versus 2019 PAP adherence for weeks 26 to 30 seems to be more the consequence of the 2019 heat wave rather than the consequence of the COVID 2019 pandemic. Other circumstantial events, such as New Year celebrations, also impact PAP adherence on a whole population basis. In our study population, the last 7 days of either year are associated with a fall in both PAP adherence and the percentage of adherent patients was lower in 2020 (not only because of a curfew but also because of a French state policy not to celebrate the New Year).

As a result of these observations, the 2020 PAP adherence of our SA-population appears to be influenced by a combination of factors, including COVID-19. It can be assumed that the impact of COVID-19 on SA-patient sleep is also influenced by the country they live in, not because of the health care system characteristics for SA-patient management but rather because of cultural and sociological parameters. In our results, people still in professional activity (age < 62) could have had less social jetlag and thus improved their sleep duration and PAP adherence [13]. As an example using crowdsourced smartphone data for non SA-populations, recent publications have described an average increase in sleep duration of 4.9 to 20 min (18.3 min for France) during the initial lockdown weekdays [13–15]. During the first lockdown, in an Italian population, a gender-dependent effect of COVID 19 pandemic on sleep quality was reported [16]. In this study, additional lockdown-related stress such as taking care of children and other family duties were hypothesized to explain women’s probability of developing sleep problems.

### Limits of the study

Our population was treated in France on a long-term basis and our conclusions may not be applicable to short-term and/or other countries.

Because of the study design and data collection, we were unable to include more than three potentially explicative variables in the multivariable logistic regression analyses, and obviously important variables (such as comorbidities, residual sleepiness, and partner presence) were unavailable.

## Conclusions

During the first and second lockdowns, the COVID-19 pandemic had a clinically irrelevant impact on PAP adherence for the study population. The 2020 PAP adherence of our SA-population appears to be influenced by a combination of factors, including COVID-19. The reader should also keep in mind that our observations are limited to long-term treated patients. Future studies are needed in other countries to describe the COVID’19 pandemic impact on PAP adherence not only for long-term PAP-treated SA patients, but also for incident cases.

## Data Availability

The datasets used and/or analyzed during the current study are available from the corresponding author on reasonable request.

## Abbreviations

AHI: Apnea–Hypopnea Index
iAHI: Initial Apnea–Hypopnea Index
MCID: Minimal clinically important difference
MLRA: Multivariable logistic regression analyses
PAP: Positive Airway Pressure
SA: Sleep-Apnea
